# A modified ‘NanoSuit®’ preserves wet samples in high vacuum: direct observations on cells and tissues in field-emission scanning electron microscopy

**DOI:** 10.1098/rsos.160887

**Published:** 2017-03-01

**Authors:** Yasuharu Takaku, Hiroshi Suzuki, Hideya Kawasaki, Isao Ohta, Daisuke Ishii, Satoshi Hirakawa, Takami Tsutsui, Haruko Matsumoto, Sayuri Takehara, Chinatsu Nakane, Kana Sakaida, Chiaki Suzuki, Yoshinori Muranaka, Hirotoshi Kikuchi, Hiroyuki Konno, Masatsugu Shimomura, Takahiko Hariyama

**Affiliations:** 1 Department of Biology, Hamamatsu University School of Medicine, Higashi-ku, Hamamatsu 431-3192, Japan; 2 Department of Chemistry, Hamamatsu University School of Medicine, Higashi-ku, Hamamatsu 431-3192, Japan; 3 Department of Regenerative and Infectious Pathology, Hamamatsu University School of Medicine, Higashi-ku, Hamamatsu 431-3192, Japan; 4 Laboratory for Ultrastructure Research and Research Equipment Center, Hamamatsu University School of Medicine, Higashi-ku, Hamamatsu 431-3192, Japan; 5 Department of Dermatology, Hamamatsu University School of Medicine, Higashi-ku, Hamamatsu 431-3192, Japan; 6 Second Department of Surgery, Hamamatsu University School of Medicine, Higashi-ku, Hamamatsu 431-3192, Japan; 7 Life Science and Applied Chemistry, Graduate School of Engineering, Nagoya Institute of Technology, Gokiso-cho, Showa-ku, Nagoya 466-8555, Japan; 8 Departments of Bio- and Material Photonics, Chitose Institute of Science and Technology, Hokkaido 066-8655, Japan

**Keywords:** field-emission scanning electron microscope (FE-SEM), *high vacuo*, living specimen, surface shield effect, NanoSuit®

## Abstract

Although field-emission scanning electron microscopy (FE-SEM) has proven very useful in biomedical research, the high vacuum required (10^−3^ to 10^−7^ Pa) precludes direct observations of living cells and tissues at high resolution and often produces unwanted structural changes. We have previously described a method that allows the investigator to keep a variety of insect larvae alive in the high vacuum environment of the electron microscope by encasing the organisms in a thin, vacuum-proof suit, the ‘NanoSuit®'. However, it was impossible to protect wet tissues freshly excised from intact organisms or cultured cells. Here we describe an improved ‘NanoSuit' technique to overcome this limitation. We protected the specimens with a surface shield enhancer (SSE) solution that consists of glycerine and electrolytes and found that the fine structure of the SSE-treated specimens is superior to that of conventionally prepared specimens. The SSE-based NanoSuit affords a much stronger barrier to gas and/or liquid loss than the previous NanoSuit did and, since it allows more detailed images, it could significantly help to elucidate the ‘real' organization of cells and their functions.

## 1. Introduction

Field-emission scanning electron microscopes (FE-SEMs) can generate images of nanosized objects by shooting electrons at them and detecting how the electrons react. To use a beam of electrons effectively, it is necessary to evacuate the specimen chamber to a high vacuum level (10^−3^ to 10^−7^ Pa) to prevent scattering by molecules in the air. In this vacuum living organisms die quickly of dehydration. To avoid tissue damage and to stabilize structures for SEM, biological specimens need to be chemically fixed prior to undergoing dehydration, freeze drying and coating with a thin layer of metal [1]. These complex procedures preclude the direct observation of living tissue and often produce unwanted structural changes even in fixed tissue. In these procedures, the drying process causes the most serious damage to the specimen, because approximately 70–80% of living tissue is water. Therefore, researchers have tried to modify SEM procedures to allow lower vacuum levels, as in low-vacuum scanning electron microscopy or by use of an environmental scanning electron microscope [2–5]. All such methods, however, resulted in inferior resolution and, thus, less information.

We have now found a way to keep multicellular organisms alive in the high vacuum of an electron microscope by encasing them in a thin, vacuum-proof suit, the ‘NanoSuit®’ [6]. The basic technique is to use the natural extracellular substance (ECS) covering some organisms or to add a substance mimicking the ECS (e.g. 1% aqueous solution of the surfactant polyoxyethylene sorbitan monolaurate; TW 20) and to polymerize these substances by plasma or electron beam irradiation. Since the NanoSuit was not only flexible but also dense enough to keep the living organisms' bodily gases and liquids from evaporating (a property that we call the ‘surface shield effect’), it works like a miniature space suit.

Although the TW 20-based NanoSuit protected some organisms in the SEM, it was unable to protect isolated tissues excised from intact organisms and cultured cells. To overcome this limitation, we have modified the technique and developed a new solution, which enables FE-SEM observations on wet tissues and/or cultured cells. The new NanoSuit allows high-resolution images to be taken of wet specimens, both living and fixed, and could significantly help to elucidate the ‘real’ organization of cells and their functions.

## 2. Material and methods

### 2.1 Experimental organisms

Male mice (BALB/c background, 20–25 g) were housed at the animal facility of Hamamatsu University School of Medicine. Mice were kept in plastic cages with free access to food and fresh water in a room with controlled temperature (22–24°C) and light (12 h/12 h light/dark cycle) at the experimental animal facility until use. Mice, 8 weeks of age, were subjected to a chronic aseptic peritonitis model as previously described [7]. Briefly, peritoneal inflammation was induced by intra-peritoneal injection of thioglycollate medium (BD Biosciences, Franklin Lakes, NJ) (5 mg per cavity in 500 µl of sterile saline) once every 3 days up to 3 weeks. All experimental procedures were performed according to The Committee on Ethical Use of Laboratory Animals of Hamamatsu University School of Medicine.

Mouse embryonic fibroblast (MEF) cells were prepared from 12.5-day-old embryos of BALB/c mice (SLC Japan, Hamamatsu, Japan). Neonatal human dermal fibroblast was purchased from Lonza (Walkersville, MD, USA). Both of the cell lines were grown in DMEM containing penicillin (100 units ml^−1^), streptomycin (50 µg ml^−1^) and 10% fetal bovine serum on the glass.

The Smith strain of MCMV was passaged in MEFs. Infectious supernatants from infected MEF cultures were made cell-free by centrifugation at 3000*g* for 20 min at 16°C. The supernatants were then ultracentrifuged for 40 min at 70 000*g*. Pellets containing virions and other particles were resuspended in 1 ml of Tris-buffered saline and transferred onto a preformed linear sorbitol gradient (25–70%), which was ultracentrifuged at 70 000*g* for 60 min. The virion-containing band was harvested with a syringe and the virions were washed and pelleted by additional ultracentrifugation at 70 000*g* for 40 min. The pellet was resuspended in phosphate-buffered saline and stored at −80°C until infection experiments.

Resected stomach tissue containing a malignant tumour was fixed in 10% neutral-buffered formalin. For SEM observation 3–5 mm thick tissue slices were cut with a scalpel. The samples included the border between malignant and normal tissue.

### 2.2. Microscopy

Field-emission scanning electron microscopy was carried out with a JEM-7100F (JEOL) and a Hitachi S-4800 instrument operated at an acceleration voltage of 1.0 kV. The vacuum level of the observation chamber was 10^−3^ to 10^−7^ Pa. The detector for secondary electrons was a mixture of signals from upper and lower detectors. Other details are as follows; working distance: 8 mm, aperture size: φ 100 µm, scan speed: each beam is 10–15 frames s^−1^. Transmission electron microscopy (TEM) observations were carried out using a JEM-1220 (JEOL) at an acceleration voltage of 120 kV.

### 2.3 Preparation of surface shield enhancer solutions and sample preparation for the FE-SEM observations

The newly developed surface shield enhancer (SSE) was used in all experiments and consisted of sucrose (5 g), fructose (5 g) and sodium chloride (5 g) dissolved in distilled water (500 ml), to which were then added under further stirring citric acid (1.25 g) and sodium glutamate (0.05 g) (pH 7.4). This aqueous solution and glycerine were mixed in a ratio of 1 : 2.

To form the NanoSuit, the specimens were dipped for 1 min into the SSE solution and blotted briefly thereafter on dry filter paper to remove excess solution. Specimens were then directly introduced into the SEM where a NanoSuit formed following irradiation by the electron beam. Alternatively, a NanoSuit was formed by pre-irradiating specimens with plasma as follows: the metal-emitter from a standard ion-sputtering device (JFC-1100, JEOL) was removed, so that the plasma ions produced within the chamber were derived from the remaining gas molecules in the chamber. Specimens were irradiated with plasma inside this device for 3 min at a vacuum level of *ca* 1.0 Pa and 1.0 kV DC (8.0 mA) at room temperature.

### 2.4 Weight loss experiment for tissues excised from intact organisms

To remove excess water remaining on the surface of the specimens, tissues, untreated and treated with SSE or Tween 20 solution [6], were exposed to low vacuum (*ca* 10 Pa) for 1 min 30 s, and then weighed for the first time. During this period, treated specimens were irradiated by plasma to construct the NanoSuit. After the SEM observations, specimens were weighed a second time. The difference before and after observations is indicated as a percentage.

### 2.5 Preparation for standard scanning and transmission electron microscopy

For standard SEM observation, samples were prefixed with 4% glutaraldehyde in 0.1 M cacodylate buffer (pH 7.4) and postfixed in 1% OsO_4_ in the same buffer. The specimens were then dehydrated, freeze dried (JFD300, JEOL) and ultra-thin coated with OsO_4_ (PMC-5000, Meiwa). For TEM to observe the surface fine structure of the samples, specimens were prefixed in 2% glutaraldehyde and 2% paraformaldehyde in 0.1 M cacodylate buffer (pH 7.4), and then postfixed in 1% OsO_4_ in the same buffer. The dehydrated specimens were embedded in an Epon–Araldite mixture. Ultra-thin sections (approximately 70 nm) were cut vertical to the surface of the sample. Sections were stained with 2% uranyl acetate followed by 0.4% lead citrate for 5 min each. To make the SSE-based NanoSuit visible, 10% platinum blue (Nisshin EM) was added to the SSE solution used to treat samples (figure 3).

## 3. Results

**Figure 1.**
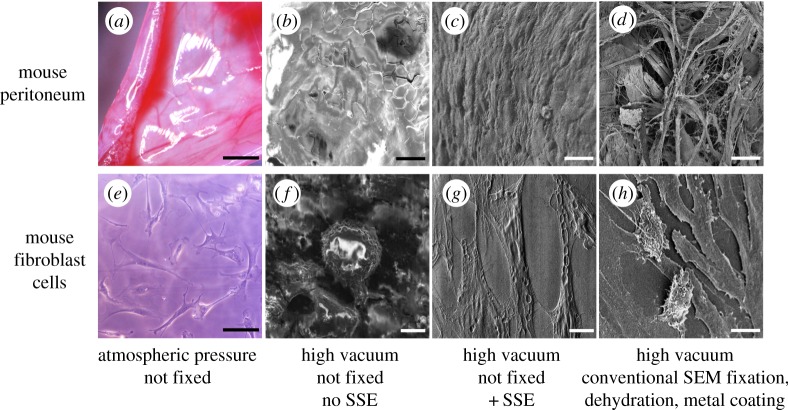
Observations of excised mouse peritoneum (top) and mouse embryonic fibroblasts (bottom) by light microscopy (*a*,*e*) and scanning electron microscopy (SEM) (*b*–*d*, *f*–*h*). Scanning electron micrographs compare specimens untreated (*b*,*f*), treated with the SSE solution (*c*,*g*) and prepared by conventional methods for SEM (*d*,*h*). Scale bars, 5 mm (*a*), 10 µm (*b*–*d*, *f*–*h*), 30 µm (*e*).

We introduced numerous living tissues and cells into the SEM to see how they changed under high vacuum (10^−3^ to 10^−7^ Pa). Figure 1 shows typical results for the peritoneum of mouse and for mouse fibroblast cells: all the untreated specimens dried up rapidly and their structural integrity was completely destroyed in the FE-SEM (figure 1*a*,*b* and *e*,*f*). The specimens also showed electrostatic charging, which prevents satisfactory imaging (figure 1*b*,*f*). We have reported previously that a NanoSuit made of 1% TW 20 prevents dehydration and electrostatic charging as long as the organisms are alive [8,9]. However, when freshly isolated peritoneum was pre-treated with the 1% TW 20, charging commenced within a few minutes, suggesting that the tissue was not protected effectively by the TW 20-based NanoSuit (electronic supplementary material, figure S1).

To protect the integrity of tissues and single cells under high vacuum, numerous combinations of solutions were tested [10]. The best results were obtained with a new SSE solution, which is a mixture of glycerine and electrolytes (see Material and methods). To test the barrier properties of the NanoSuit based on this solution, tissues and cell cultures were dipped in SSE solution and then exposed to an electron beam or plasma irradiation. When these specimens were subsequently introduced into the FE-SEM they showed no observable change in appearance (figure 1*c*,*g*) suggesting that the SSE prevented desiccation of the samples. To quantify the ability of the SSE-based NanoSuit to act as a barrier for gas and/or liquid, the weight loss of peritoneum samples was measured after 30 min observation under high vacuum of the SEM. The results in table 1 show that untreated specimens and specimens treated with the TW 20 solution lost roughly 50% of their weight after 30 min whereas specimens treated with the SSE solution showed only 9% weight loss. Thus, the NanoSuit formed with the SSE solution is an effective barrier to water loss. All of the SSE-treated specimens could be observed in the SEM with good resolution and no electrostatic charging for up to 60 min. Specimens that we have tested include whole mammalian organs, excised mammalian tissue samples and cell cultures (e.g. cancer cell lines).

**Table 1.** Weight loss of unfixed and fixed tissue samples after SEM observation.

weight loss in samples of mouse peritoneum treated with TW 20 and SSE and observed in SEM (*N* = 10)	
no treatment	58.3%
+ SSE	38.4%
+ SSE + plasma	8.9%
+ TW20 + plasma	48.6%
weight loss in samples of normal and cancerous stomach tissue and observed in SEM (*N* = 10)	
no treatment	54.2%
+ SSE + plasma	10.1%

**Figure 2.**
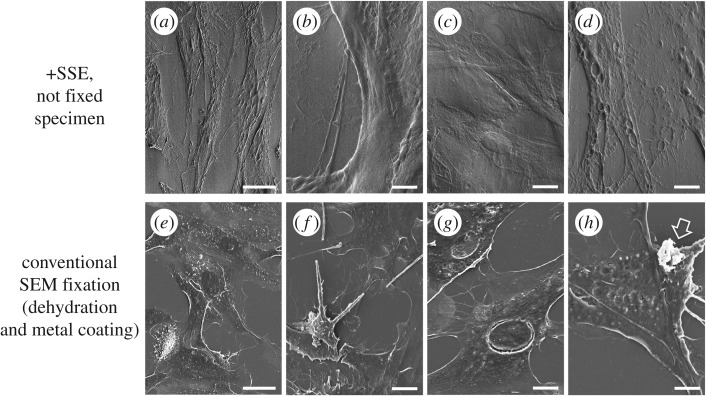
Comparison of images of human fibroblast treated with the SSE solution (*a*–*d*) and prepared by traditional methods (*e*–*h*). Fibres of the cells (*b*,*f*), nucleus (*c*,*g*), high magnifications of the cell surface (*d*,*h*). In traditional methods, some cells show protrusions on the surface (arrow in *h*). Scale bars, 20 µm (*a*,*e*), 10 µm (*b*,*c,f,g*), 5 µm (*d*,*h*).

In addition to the apparent barrier effect, we found that the fine structure of the specimens treated with SSE solution was completely different from that of conventionally prepared, i.e. fixed and metal coated, specimens. Following conventional preparation, shrinkage of tissues and cells was inevitable owing to dehydration (figures 1*d*,*h*, 2*e*–*h*). By comparison, the overall morphology of SSE-treated specimens seemed well preserved and intact (figures 1*c*,*g*, 2*a*–*d*).

**Figure 3.**
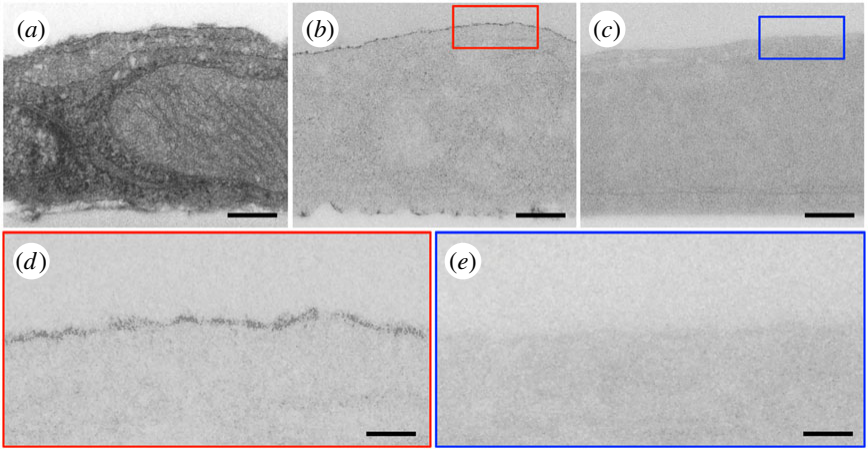
Imaging the SSE NanoSuit. (*a*,*b*) TEM images of cells treated with SSE and then fixed and sectioned for TEM. The SSE solution contained platinum blue to increase contrast of the NanoSuit. The SSE NanoSuit is about 10 nm thick. (*c*) TEM image of control cells not treated with SSE but fixed and sectioned as in *a* and *b*. The TEM section in *a* is similar to *b* but was stained with 2% uranyl acetate and 0.4% lead citrate to reveal subcellular structures. The red and blue squares in *b, c* are shown at high magnifications in *d*, *e*, respectively. Scale bars, 300 nm (*a*–*c*), 100 nm (*d*,*e*).

To investigate the structure of the SSE-based NanoSuit we fixed and stained cultured cells treated with SSE and plasma irradiation for TEM (figure 3). Cross-sections showed that specimens produced an extra layer (less than 10 nm thickness) over their surface (figure 3*a*,*b*,*d*). Since no such layer was detected in specimens, which were not treated with the SSE (figure 3*c*,*e*), we conclude that this ‘surface shield’ is what protects specimens from desiccation in the SEM. The SSE-based surface shield is much thinner than the TW 20-based NanoSuit (50–200 nm) that we previously developed [9]. As a consequence it is possible to image surface structures at much higher resolution.

**Figure 4.**
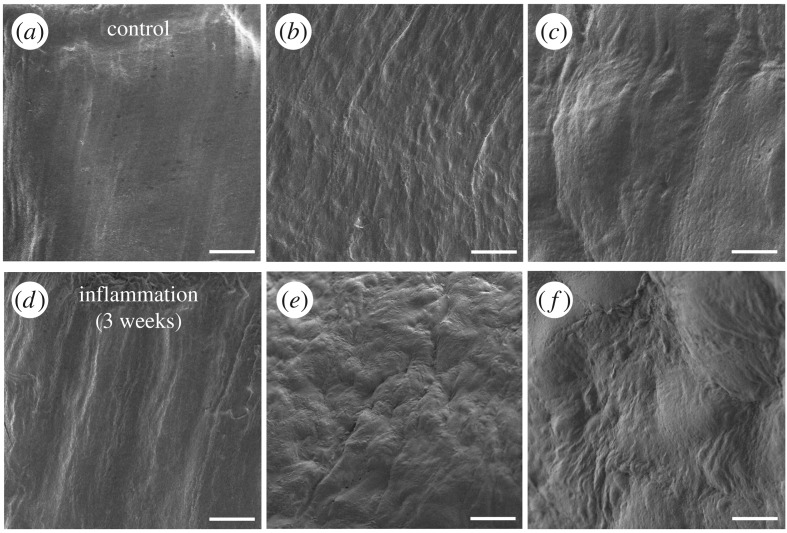
Control living peritoneum in mouse (*a*–*c*) and images of inflamed specimen with adjuvand for 3 weeks (*d*–*f*). All specimens were treated with the SSE solution, without chemical fixation and dehydration. Scale bars, 200 µm (*a*,*d*), 20 µm (*b*,*e*), 2 µm (*c*,*f*).

To determine the effectiveness of the new SSE-based NanoSuit to identify morphological features at high magnification, we investigated the difference between normal and inflamed mouse peritoneum (figure 4*a*–*c*). Using conventional electron microscopic methods, it was difficult to distinguish if the observable damage to tissue was caused by inflammation or by the fixation and dehydration processes (data not shown). However, specimens treated with SSE appeared normal and showed smooth surfaces when compared with tissues inflamed with adjuvant for 3 weeks (figure 4*d*–*f*). This suggests that the morphological differences are the result of the inflammation.

**Figure 5.**
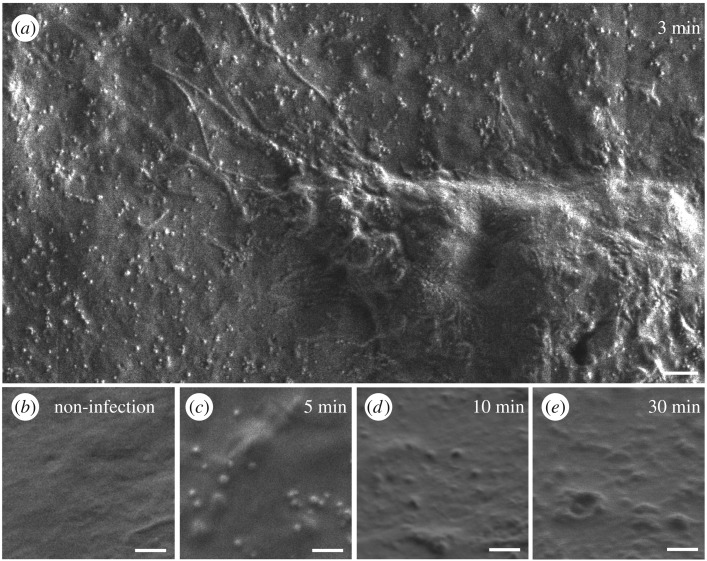
SEM images of the cell surface of mouse fibroblasts infected with cytomegalovirus. Cells were treated with SSE and imaged directly in SEM. (*a*) Infected cell after 3 min. (*b*) Uninfected cell. (*c*–*e*) Infected cells after 5, 10 and 30 min, respectively. Scale bars, 2 µm (*a*), 500 nm (*b*–*e*).

To further investigate the ability of the SSE NanoSuit to improve high-resolution imaging of living cells, we made direct observations of an animal virus infecting cells. Mouse fibroblasts infected with cytomegalovirus were treated with the SSE solution and introduced into the SEM. Images taken 3–5 min after infection showed large numbers of virus particles attached to the cell surface (figure 5*a*,*c*). Uninfected cell had no such particles on the cell surface (figure 5*b*). Interestingly, we observed that the appearance of the cell surface in the SEM changed with time. After 3–5 min virus particles were clearly visible on the cell surface but after 10 and 30 min the virus particles appeared to be taken up by endocytosis while being observed in the SEM (N.B. the temperature in the SEM chamber is 20°C) (figure 5*d*,*e*). A similar but more rapid change in cell surface morphology was observed in infected cells, which were fixed at various times after infection and then treated with SSE and observed in the SEM (electronic supplementary material, figure S2*b–d*). Although the specimens in electronic supplementary material, figure S2*b*–*d* were fixed with 4% glutaraldehyde, they remained ‘wet’ even in high vacuum of the SEM owing to the presence of the SSE NanoSuit.

**Figure 6.**
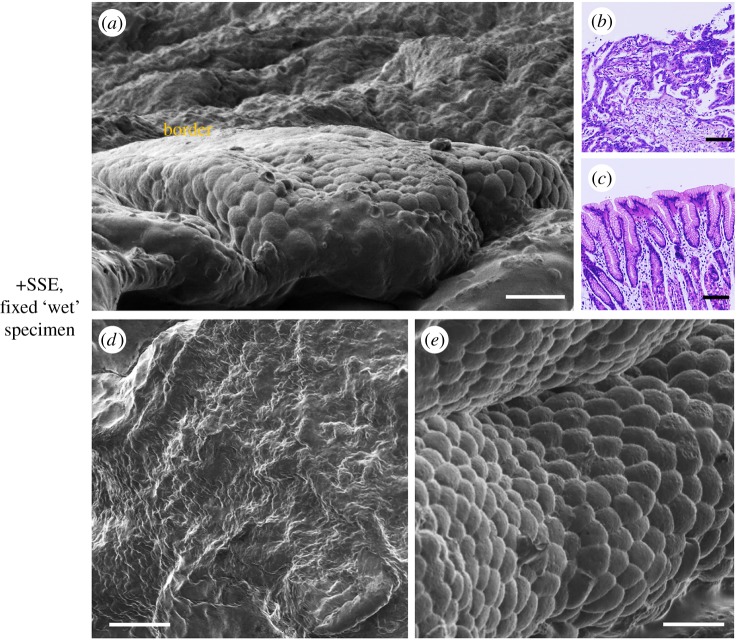
Comparison of normal and cancerous stomach tissue protected with SSE-based NanoSuit and observed directly in SEM (*a*) or stereo dissecting images of haematoxylin and eosin (H&E); cancerous (*b*) and normal tissue (*c*) in the same specimen. High magnification images of cancerous (*d*) and normal (*e*) regions protected with SSE-based NanoSuit in SEM. Scale bars, 20 µm (*a*), 0.2 mm (*b*,*c*), 10 µm (*d*,*e*).

To explore the effect of the SSE-based NanoSuit for ‘wet fixed specimens', observations were carried out on chemically fixed pathology samples. Figure 6 shows images of a surgical explant of human stomach wall including areas of cancerous and normal tissue. Morphological features were compared using two different preparations: specimens treated with SSE solution and imaged directly in the SEM (figure 6) and specimens prepared by conventional methods for SEM observation (fixation, freeze drying and metal coating) (electronic supplementary material, figure S3). Specimens treated with SSE solution (figure 6*a*,*d*,*e*) appeared intact. By comparison, fixed specimens prepared with conventional metal coating for SEM showed obvious structural damage (electronic supplementary material, figure S3). Consistent with these results, specimens treated with SSE showed little weight loss following 30 min exposure to the SEM whereas untreated samples showed major weight loss in the SEM (table 1).

To identify cancerous and normal tissue in the explants, we compared the results of haematoxylin and eosin (H&E) staining (figure 6*b*,*c*) with the electron microscopic imaging (figure 6*a*,*d*,*e*). The SSE-treated specimens preserved fine structures and remained ‘wet’ during the SEM observation and normal and cancerous tissue showed clear differences in morphology. Cancerous cells had a flat surface (figure 6*d*) compared with clearly rounded surface profiles of normal cells (figure 6*e*). These results indicate that the SSE NanoSuit played a significant role as a barrier preventing desiccation in the high vacuum. In summary, covering the surface of fixed tissue explants with an SSE-based NanoSuit permits rapid and easy identification of abnormal and normal regions in tissue.

## 4. Discussion

To maintain life under natural conditions, water, which possesses many unique properties and plays an irreplaceable role in organisms, is an essential chemical. Thus, water limitation is one of the harshest stressors in an extreme environment, *viz.* a high vacuum. We previously found that after modification of material on the surface of organisms through exposure to an electron beam [11] or plasma ionization [12], i.e. conditions known to enhance polymer formation, some treated animals survived under the high vacuum condition of the FE-SEM owing to the protective properties of what we termed the NanoSuit. The NanoSuit allows more sophisticated observation methods for studying living organisms in a FE-SEM.

In our earlier report, we used TW 20 to form the NanoSuit on the living organisms [6,8–10]. In whole animals, which were successfully protected with a TW 20-based NanoSuit, the outer surface was covered with epithelium or cuticle. By contrast, excised tissues or single cells do not have such a protective cover, so that an alternative barrier was needed. In the present experiment, we used an SSE solution with glycerine as a main component. Since glycerine is strongly hygroscopic, it has been used as a humectant in cosmetics [13]. To increase the barrier effect, we have combined glycerine with electrolytes and polymerized a thin liquid film over the sample in order to prepare a NanoSuit. In some cases this treatment yielded an effective NanoSuit and permitted imaging in the SEM (figures 1*c*,*g*, 2*a*–*d*, 4, 5 and 6*a*,*d*,*e*, and the electronic supplementary material, figure S2). The SSE constitutes a very effective diffusion barrier (table 1) and it is very thin (less than 10 nm) (figure 3*a*,*b*,*d*). The SSE-based surface shield is much thinner than the TW 20-based NanoSuit (50–200 nm) that we previously developed [9]. As a consequence it is possible to image surface structures at much higher resolution. Since glycerine alone is unlikely to polymerize to form a stable NanoSuit, it seems likely that it interacts with protein/proteoglycan on the surface of cells and tissues to form a stabilizing polymer coat after plasma irradiation or exposure to the electron beam in the SEM.

There have been several previous attempts to adapt SEM for the observation of wet samples. Thiberge *et al.* used polyimide or silicon nitride membranes to protect the sample from the vacuum [14]. However, this method required the use of high acceleration voltages (15–30 kV) to penetrate the relatively thick membranes. The intense radiation of the electron beam during high magnification imaging was sufficient to cause damage to the specimens. A related technique for imaging wet tissue in SEM has recently been described by Wojcik *et al*. [15]. This method covers tissue specimens with a monolayer of graphene. In addition to being technically challenging, these methods appear only to work successfully with fixed tissue samples. By contrast, our SSE-based NanoSuit method can be applied to both living specimens (figures 1*c*,*g*, 2*a*–*d*, 4 and 5) and to fixed tissue (figure 6*a*,*d*,*e*; electronic supplementary material, figure S2*b*–*d*) and requires only use of low voltage electrons (1 kV). Imaging occurs in a hydrous/wet state closely approximating the natural condition. Moreover, even after SSE treatment and exposure to the high vacuum in the SEM, some cells survived and could be re-cultured when they were returned to atmospheric pressure and placed in culture medium (Takaku Y *et al.*, in preparation). Our results suggest that, to protect cells for long periods under *high vacuo* conditions, very effective barriers against the extreme environment need to be devised, and at the same time improvements in the SEM instrument to accommodate living organisms will be necessary. This ongoing progress in electron microscopy is starting to have a significant impact on our understanding of the subcellular world.

## Supplementary Material

Figure S1

## Supplementary Material

Figure S2

## Supplementary Material

Figure S3

## Data accessibility

Our data can be found in the electronic supplementary material.
