# Supplementary figures and images for: A modified ‘NanoSuit®’ preserves wet samples in high vacuum: direct observations on cells and tissues in field-emission scanning electron microscopy
Source: R Soc Open Sci. 2017 Mar 1;4(3):160887. doi: 10.1098/rsos.160887 (PMC5383832; doi:10.1098/rsos.160887)

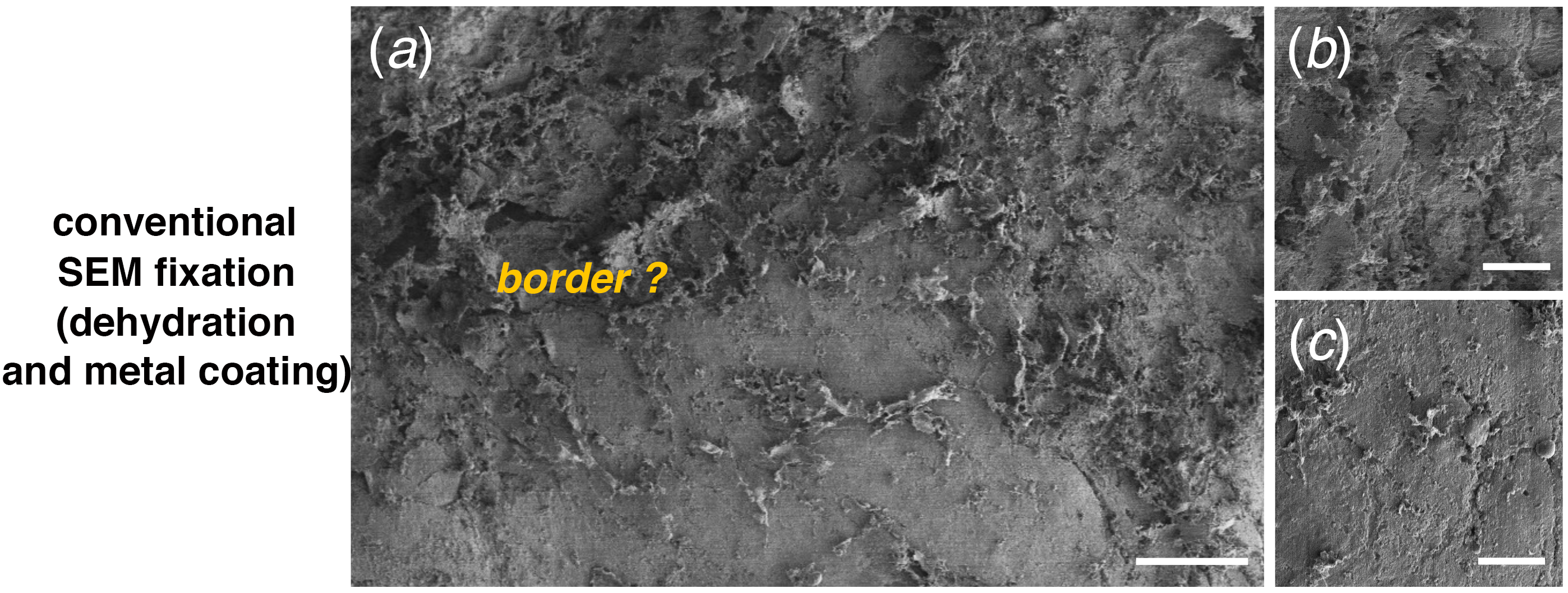

Supplement: Figure S1 [file rsos160887supp1.tif]

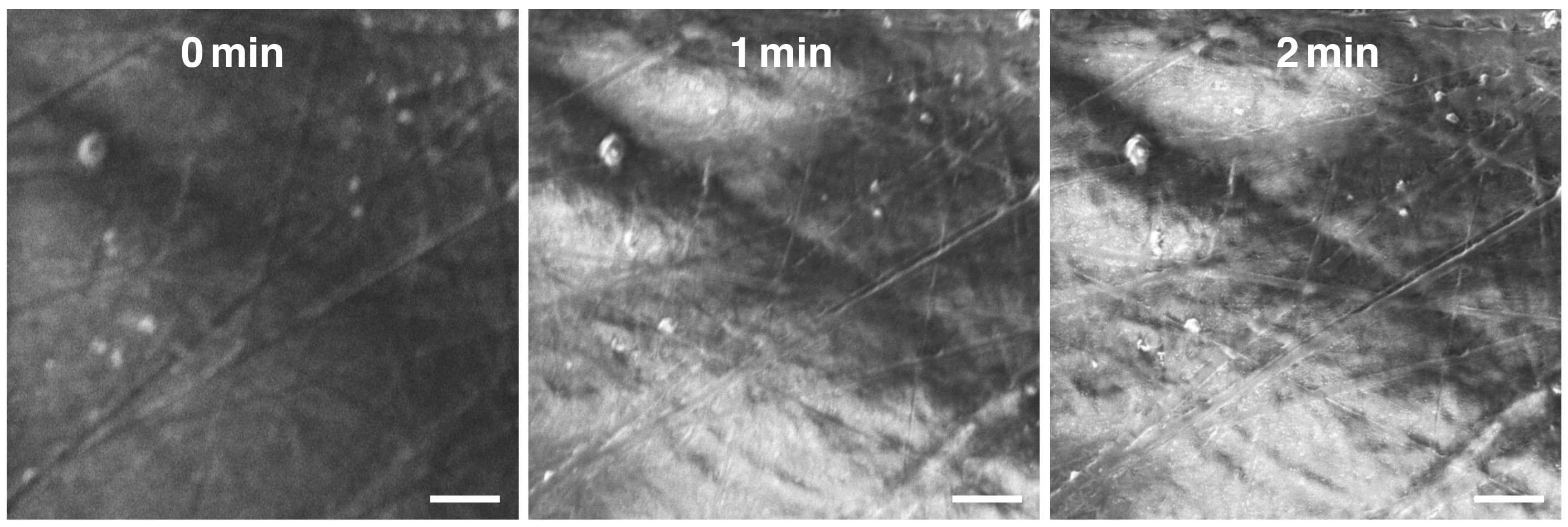

Supplement: Figure S2 [file rsos160887supp2.tif]

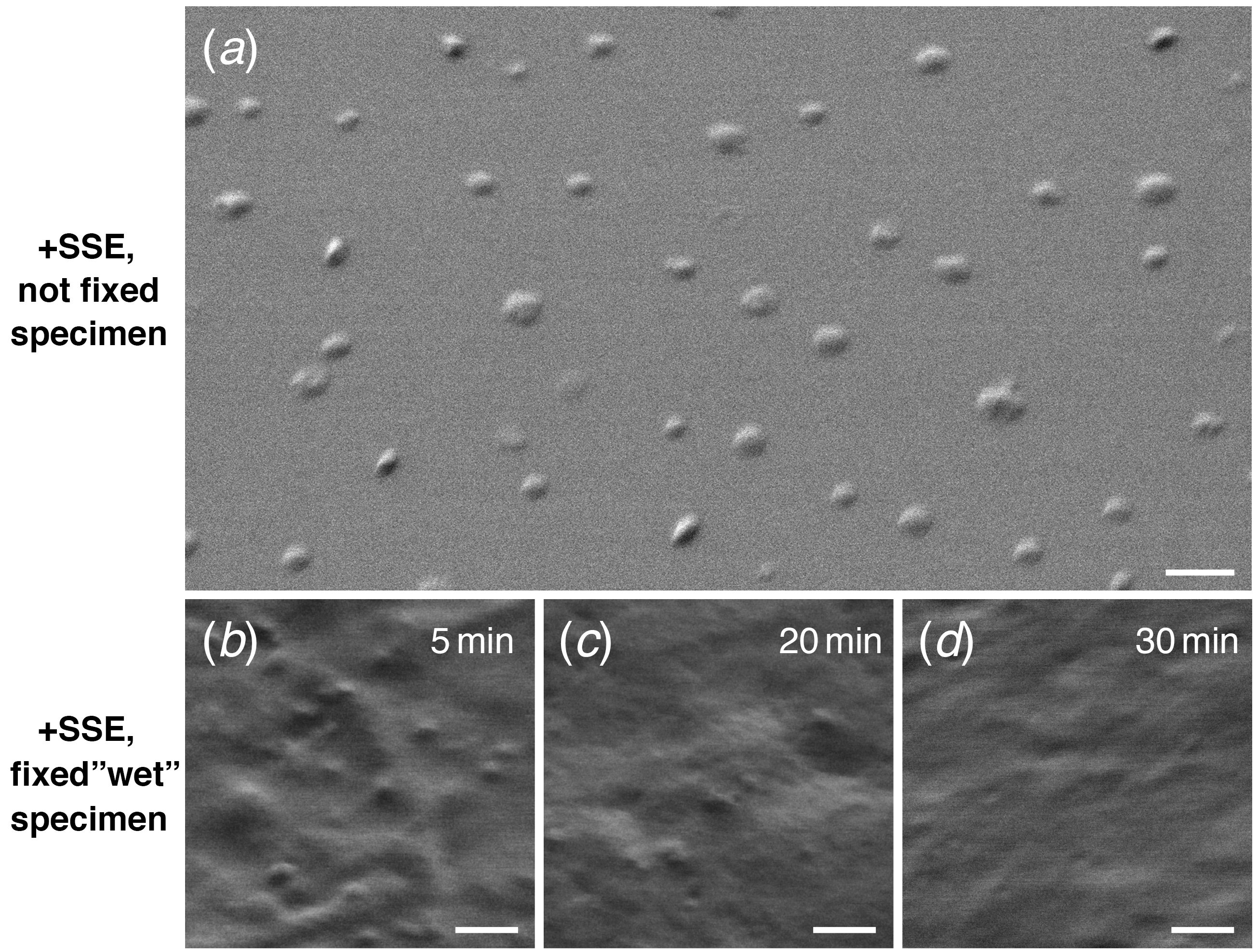

Supplement: Figure S3 [file rsos160887supp3.tif]
